# Pine invasions in treeless environments: dispersal overruns microsite heterogeneity

**DOI:** 10.1002/ece3.1877

**Published:** 2016-01-08

**Authors:** Aníbal Pauchard, Adrián Escudero, Rafael A. García, Marcelino de la Cruz, Bárbara Langdon, Lohengrin A. Cavieres, Jocelyn Esquivel

**Affiliations:** ^1^ Laboratorio de Invasiones BiológicasFacultad de Ciencias ForestalesUniversidad de ConcepciónVictoria 631, Casilla 160‐CConcepciónChile; ^2^ Institute of Ecology and Biodiversity (IEB)Las Palmeras 3425 Ñuñoa, Casilla 653SantiagoChile; ^3^ Biodiversity and Conservation UnitDepartment of SciencesKing Juan Carlos Universityc/Tulipán s/n.28933MóstolesMadridSpain; ^4^ Programa Conservación de FloraBioforest SACamino a Coronel km 15 S/NConcepciónChile; ^5^ Departamento de BotánicaFacultad de Ciencias Naturales y OceanográficasUniversidad de ConcepciónCasilla 160‐CConcepciónChile

**Keywords:** Invasibility, *Pinus contorta*, plant–plant interactions, propagule pressure, spatial patterns, tree invasions

## Abstract

Understanding biological invasions patterns and mechanisms is highly needed for forecasting and managing these processes and their negative impacts. At small scales, ecological processes driving plant invasions are expected to produce a spatially explicit pattern driven by propagule pressure and local ground heterogeneity. Our aim was to determine the interplay between the intensity of seed rain, using distance to a mature plantation as a proxy, and microsite heterogeneity in the spreading of *Pinus contorta* in the treeless Patagonian steppe. Three one‐hectare plots were located under different degrees of *P. contorta* invasion (Coyhaique Alto, 45° 30′S and 71° 42′W). We fitted three types of inhomogeneous Poisson models to each pine plot in an attempt for describing the observed pattern as accurately as possible: the “dispersal” models, “local ground heterogeneity” models, and “combined” models, using both types of covariates. To include the temporal axis in the invasion process, we analyzed both the pattern of young and old recruits and also of all recruits together. As hypothesized, the spatial patterns of recruited pines showed coarse scale heterogeneity. Early pine invasion spatial patterns in our Patagonian steppe site is not different from expectations of inhomogeneous Poisson processes taking into consideration a linear and negative dependency of pine recruit intensity on the distance to afforestations. Models including ground‐cover predictors were able to describe the point pattern process only in a couple of cases but never better than dispersal models. This finding concurs with the idea that early invasions depend more on seed pressure than on the biotic and abiotic relationships seed and seedlings establish at the microsite scale. Our results show that without a timely and active management, *P. contorta* will invade the Patagonian steppe independently of the local ground‐cover conditions.

## Introduction

Biological invasions are magnificent natural experiments for the study of spatially explicit phenomena such as dispersal, colonization, range expansion, and population dynamics from global to local scales (Richardson and Rejmánek 2004; Pauchard and Shea 2006). Data on plant invasions at large temporal and spatial scales, available in a variety of sources, such as herbaria vouchers and quarantine office records, have delivered insightful trends for ecology and biogeography (Sagarin and Pauchard 2012; Kueffer et al. 2013). However, mechanisms involved in plant invasions at finer spatial scales have not yet being fully explored and understood.

At local scales, ecological processes driving plant invasions are expected to produce a spatially explicit pattern, with recruited individuals distributed according to an intensity function (i.e., with changes in the local density of the invader), which can be related to critical demographic processes. In fact, local plant invasion patterns depend on the simultaneous action of two basic processes, which produce a profound spatial signal on the invader's recruitment (McIntire and Fajardo 2009): first, dispersal and arrival of seed or propagules and, second, the complex interaction of them with the ground surface components (i.e., environmental heterogeneity) (De la Cruz et al. 2008). Seed arrival at a point is mainly dependent of the magnitude of the seed source (i.e., number of seeds dispersed), the distance to the seed source and of seed functional traits determining the dispersal kernel (Nathan and Muller‐Landau 2000).

Ground heterogeneity may have important effects on invasion through a complex sequence of demographic processes such as differential seed trapping (e.g., rocks, bare, shrubs, and grasses) and the establishment of biotic interactions such as seed predation and both facilitative and competitive plant–plant interactions during germination and early seedling survival and growth (Bruno et al. 2005). It is well known that microsite characteristics, which are the sum of multiple factors including site suitability as well as biotic interactions, may become critical for the invasion process (e.g., Cavieres et al. 2005; Quiroz et al. 2011; Souza et al. 2011). So far, most suggested invasive mechanisms are related to the existence of negative interactions among native and non‐native species, such as higher competitive ability of the invader due to natural enemy releases (Blossey and Nötzold 1995), the lack of herbivores and pathogens in the invasive habitats (Keane and Crawley 2002) or the possession of allelopathic compounds (Hierro and Callaway 2003). More recently, the potential role of positive interactions for invasion has also received attention (Simberloff and Von Holle 1999; Richardson et al. 2000; Bruno et al. 2005; Rodriguez 2006; Altieri et al. 2010). Non‐native animals and plants may modify the environment in ways that favor the spread of other non‐native species and therefore, facilitating the invasion (Simberloff and Von Holle 1999; Richardson et al. 2000). In particular, positive plant–plant interactions may be important drivers of invasion in ecosystems under more stressful environmental conditions (Bruno et al. 2005; Bulleri et al. 2008).

Point pattern analysis (Wiegand and Moloney 2004) is a powerful tool to determine the relative contribution of both dispersal and the invaded community heterogeneity to the realized pattern of a plant invasion. An insightful approach would be considering that both factors (seed dispersal and ground heterogeneity) control the intensity of an inhomogeneous Poisson process that determines the spatial distribution of individuals (Wright 2002; Wiegand et al. 2007; Murrell 2009). This statistical could be easily interpreted in ecological terms. For example, ground surface heterogeneity could cause higher densities of recruits in favorable microsites including beneath nurse plants, or higher mortality and poor plant growth in less favorable microsites (Getzin et al. 2008; Murrell 2009). On the other hand, areas where the seed rain is higher would have a larger density of invader recruits. In addition, it could be also possible to reveal the importance of these two mechanisms or shifts among them along the invasion process by just analyzing the changes in the spatial pattern over time (Getzin et al. 2008; Murrell 2009), or when no repeated censuses are available, just comparing the spatial distribution of individuals of different age (Getzin et al. 2008; Zhu et al. 2010; Bagchi et al. 2011). As a corollary, using adequate null models based on processes of biological sense, we can test whether or not the observed patterns are compatible with the incidence of such causes.

Spatially explicit information is especially interesting in the case of pine invasions, a worldwide concern that demands more information for developing adequate managing tools (Richardson and Higgins 1998; Haysom and Murphy 2003). For instance, very little is known about the role of interspecific interactions in determining the invasion success of pines (Richardson 2006). Different species in the recipient community may establish different interactions with the spreading pine, with a net effect on the establishment and growth of the invasive pine that will depend upon the relative abundance of each species. These different putative effects of the resident species have never been explored in a spatially explicit context so far as a factor affecting pine invasion success and realized patterns.

**Figure 1:**
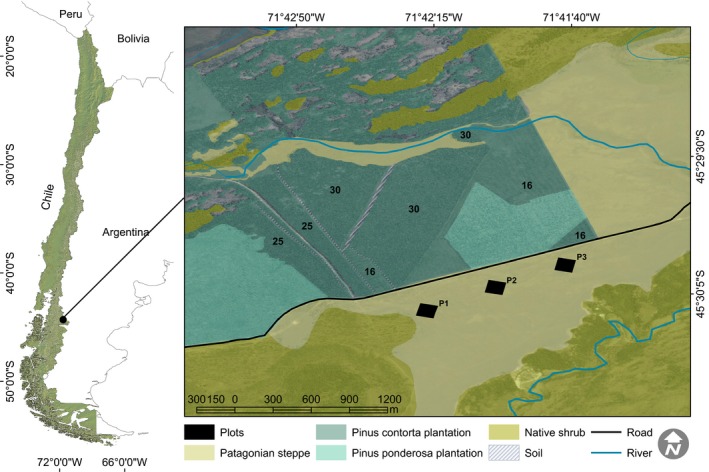
Above: Study area map overlaid in a satellite image, showing the location of the three‐one‐hectare plots and pine afforestations. Below: A panoramic view of the study area, where afforestations are in the back to the left and the invasion in the steppe in front and to the right. Notice the presence of an invasion front as well as some isolated pines (Langdon et al. 2010).

*Pinus contorta* has been recognized as a valuable model species to test several hypotheses about patterns, mechanisms, and impacts of tree invasions (Gundale et al. 2014). *P. contorta* is considered as one of the most invasive plantation trees (Rejmánek and Richardson 1996; Ledgard 2001; Gundale et al. 2014). Its invasiveness is due to its early seed production, small seed size, short time between massive seed crops (Rejmánek and Richardson 1996) and high rates of seedling growth under very different conditions (Grotkopp et al. 2002). Its invasion is consistent across temperate regions of the world (Richardson et al. 1994) and is characterized by the formation of a fringe spread with dense regeneration close to the seed source (i.e., short distance dispersal from an afforestation), and some scattered outlier trees established further than a few hundred meters from plantations (Richardson and Higgins 1998; Richardson 2001; Ledgard 2003; Langdon et al. 2010; Taylor et al. 2016) (Fig. 1).

Treeless Patagonian steppe is one of the most affected ecosystems by pine invasions on a global scale, and large afforestation projects are only worsening the problem. In fact, *P. contorta* can easily disperse and get established forming dense monospecific stands that displace the natural Patagonian steppe vegetation (Sarasola et al. 2006; Peña et al. 2008; Langdon et al. 2010).

Our aim was to determine the interplay between seed rain, using distance to the mature plantation as a proxy, and microsite heterogeneity in the spreading of *Pinus contorta* during the earliest stages of pine invasion Using three‐one‐hectare plots in the invasion front, we assessed the role of seed rain and different ground surface components at fine spatial scales to explain the observed patterns of pine recruits. In synthesis, we wanted to test whether the fine scale pattern of the invasion is dependent on the seed rain or the ground heterogeneity or a combination of both. Our research question is a priority not just to help to complete the current plant invasion paradigm, but also in applied terms to know what are the factors controlling their spread for an adequate management and to forecast the consequences of leaving these invasions uncontrolled.

## Methods

### Study species

*Pinus contorta* is an aggressive species reported as naturalized in Russia and as invader in Australia, Argentina, Chile, Ireland, New Zealand, Sweden, United Kingdom, and the United States (outside its natural range) (Haysom and Murphy 2003; Richardson 2006; Gundale et al. 2014; Taylor et al. 2016).

*Pinus contorta* native range includes the Rocky Mountains and the Pacific West of the United States and Canada, under a wide variety of climates (Despain 2001). Within its broad distributional range, this species experiences minimum temperatures between 7°C and −57°C, maximum temperatures between 27°C and 38°C, and annual precipitation varying from 250 to 500 mm. It is very prolific with mass seed crops every 1 or 3 years. Viable seeds productions starts as soon as 5–10 years. Cone productions can vary from a few hundred to a few thousands per tree (Despain 2001). Annual crops can vary from 173,000 to 790,000 seed per hectare with half or a third of them available for seedfall in those places where some portion of the trees is of the serotinous type. Where nonserotinous cone habit is prevalent, seedfall varies from 35,000 to 1.2 million seeds per hectare. The number of seeds stored is probably ten times larger than the number of seeds produced annually. The size of the seeds of *P. contorta* is small within the *Pinus* genus. Weight varies from 2.3 mg to 11.4 mg in different locations of its distributional range (Lotan and Critchfield 1990). Best conditions for germination are full sunlight and mineral soil. Temperatures between 8°C and 26°C are the most favorable, and snow‐melt moisture is required after germination (Despain 2001). Seedlings are resistant to freeze damage and very intolerant to shade. Stand density can reach thousands or even hundred thousand trees per hectare (Lotan and Critchfield 1990).

### Study area

We studied an invaded area in Coyhaique Alto (45° 30′S; 71° 42′W), at 36 km east from Coyhaique in the Aysén Region (Fig. 1). This region belongs to the Cold Steppe Ecoregion (Hepp 1996), characterized by a short growing season (Ulriksen et al. 1979) and a 6‐month period of water stress with a winter dormancy period of 9 months (Ganderats 2001). Patagonian steppe vegetation is dominated by small tussocks, mainly of *Festuca, Agrotis, Stipa, Poa,* and *Bromus* species (Hepp 1996) accompanied by some cushion‐like shrubs such as *Baccharis* spp., *Mulinum spinosum*, and *Acaena* spp. (Hepp 1996). Some of these cushion‐like species have been described as nurse plants (Nuñez et al. 1999), whereas it has been shown that adult tussock grasses can exert a strong competitive effect on woody species in Patagonia (Cipriotti et al. 2014). In the site, several afforestations with pine species were conducted since the 1980s. Main species planted in this area were *Pinus ponderosa* (1253 ha) and *Pinus contorta* (683 ha), planted between 1980 and 1999 (Fig. 1). *Pinus contorta* stands, located close to the invasion area, and thus acting as seed sources, were planted in 1981, 1986, and 1995. Together they account for 116.7 ha, with densities above 10,000 trees ha^−1^.

### Data collection

**Figure 2:**
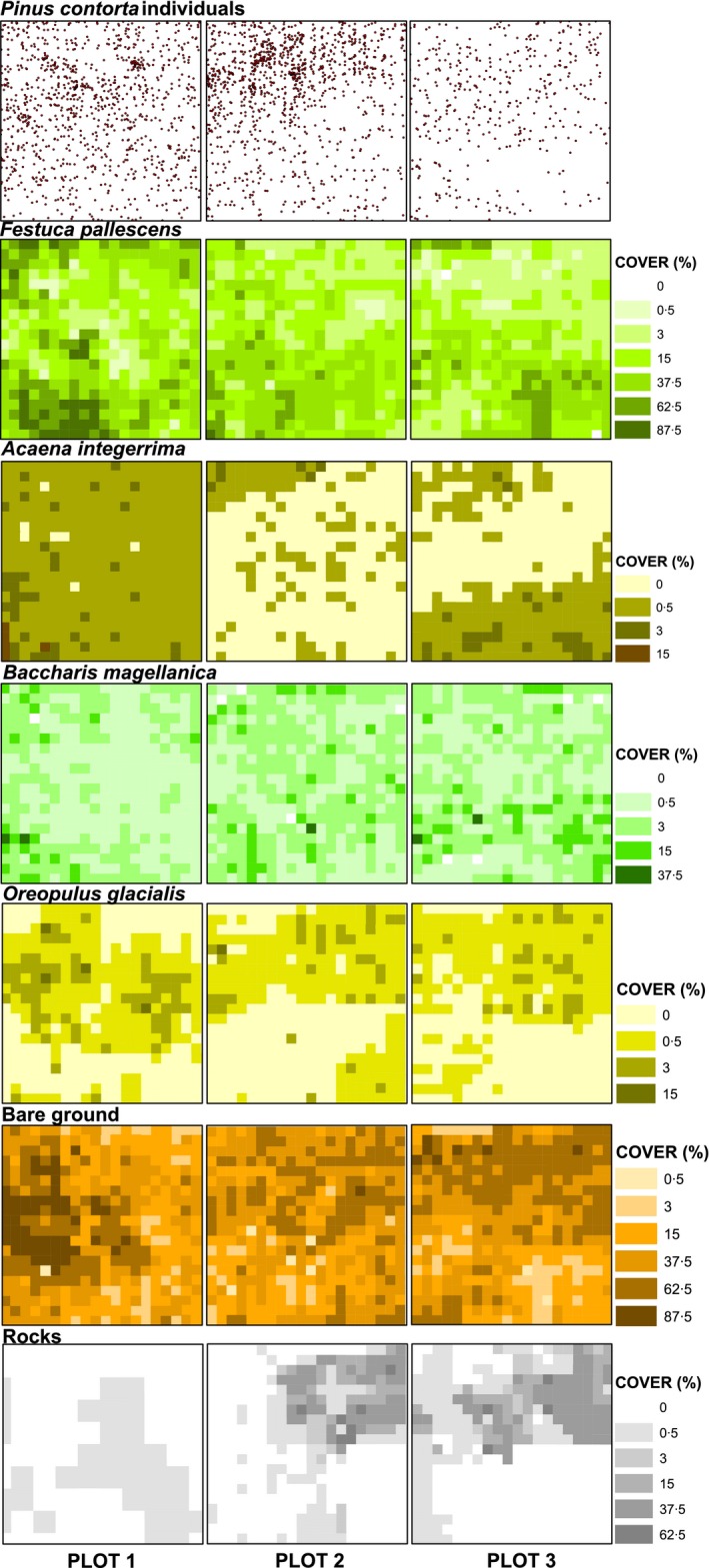
Spatial patterns recorded at the three‐one‐hectare plots (100 by 100 m) including *Pinus contorta* individual point pattern and ground covers of *Festuca pallescens*,* Acaena integerrima*,* Baccharis magellanica*,* Oreopulus glacialis*, bare ground and rocks. The upper side of each plot is the closest to the afforestations (propagule source).

In January 2011, three‐one‐hectare plots (100 by 100 m) were located at different distances of the afforestation areas in homogeneous native steppes under different degrees of pine invasion. These three plots (Fig. 1) represent a chronosequence of the invasion process (Langdon et al. 2010; Visser et al. 2014). Within each one‐hectare plot, we mapped to the nearest cm the spatial coordinates of each pine (Submetric Trimble GPS) and measured its diameter at the root collar (drc) (Fig. 2). As we are interested in the temporal pattern of the invasion, we split our global data set in two subsets: the smallest and largest pines. In each plot, pines with ln(drc) < mean (ln(drc)) – sd(ln(drc)) were classified as “small”, and those with ln(drc) > mean (ln(drc)) + sd(ln(drc)) were classified as “large” (Appendix: Table A1). Obviously, this last group includes all those pines which were the first in the invasion, whereas the small pines group corresponds with the most recently recruited. Additionally, each plot was subdivided in 400 subplots (5 × 5 m), and within each, the percentage cover of every perennial at the species level, of bare soil and rocks cover, was visually estimated (Fig. 2). Also, in each plot, we set a grid of 400 × 400 points (i.e., a point each 25 cm) and computed the shortest distance from each point to the border of the *P. contorta* afforestation plots (Fig. 1). This was performed as a surrogate of the potential seed dispersal shadow that reaches a maximum at the minimum distance of the seed source. We computed shortest distances to the set of all afforestations as a whole (“contorta”) and to each individual afforestation considering the age from the planting (i.e., “contorta16”, “contorta25”, “contorta 30”).

### Data analyses and spatial modeling

We analyzed spatial pattern of pine trees using Ripley's *K*‐function (Ripley 1976). For a homogeneous point pattern with intensity (i.e., density) *λ*,* λK*(*r*) is the expected number of points within a circle of radius *r* around an arbitrary point. We also used the inhomogeneous *K*‐function [Kinhom(*r*)], which can be defined as the expected value, for an arbitrary point *u*, of the sum of all terms 1/*λ* (*x*_*j*_) over all points *x*_*j*_ in the pattern separated from *u* by a distance less than *r,* where *λ* (*x*_*j*_) is the intensity in the location of the point *x*_*j*_ (Baddeley and Turner 2005). It reduces to the ordinary *K*‐function if *λ* is constant. For ease of visual interpretation, we used the *L*‐function, that is, the square root transformed *K*:* L*(*r*) = [*K*(*r*)/*π*]^1/2^ −* r* (Besag 1977). If the pattern is compatible with a homogeneous Poisson process (i.e., Complete Spatial Randomness), or if the pattern is inhomogeneous Poisson with intensity function *λ*(*u*), then *K* (*r*) =* Kinhom*(*r*) *= πr* ^2^ and *L*(*r*) = *Linhom*(*r*) =* *0. Both homogeneous and inhomogeneous *K*‐functions were estimated using the isotropic edge correction of Ripley (Ripley 1978).

### Test of heterogeneity

As a preliminary exploratory step, we tested the observed *L*‐function of each plot against a null model considering a homogeneous Poisson process. Large‐scale heterogeneity originates locally elevated tree densities, which leads to strong increases of the *L*‐function at larger scales (Wiegand and Moloney 2004). Significant deviations from a homogeneous Poisson process at scales *r* > 10 m are usually accepted as a confirmation of the existence of and environmental heterogeneity ruling the spatial pattern of forests trees (Wiegand and Moloney 2004; Chacón‐Labella et al. 2014). For each plot, we compared the *L*‐function computed on the pattern of pine recruits with the 3rd lowest and 3rd highest value of 99 simulations of patterns generated by a homogeneous Poisson process. As the simulation envelopes represent point‐wise tests and this approach may inflate type I error, we performed a global goodness‐of‐fit—GOF—test (Loosmore and Ford 2006) on the results of each analysis.

### Accounting for heterogeneity

As the homogeneous *L*‐functions indicated the existence of large‐scale heterogeneity in most plots, we fitted inhomogeneous Poisson models to each pine population/plot using the ppm() function of Spatstat (Baddeley and Turner 2005). This function fits the intensity of the observed point pattern as a log‐linear function of one or several covariates maximizing the likelihood (Baddeley and Turner 2000). We fitted three types of model: the “dispersal” models (using as covariates the maps of distances to *P. contorta* afforestations), the “local ground heterogeneity” models (using as covariates the maps of soil, rocks and the most abundant perennials cover), and the so‐called combined models, using both types of covariates. For each plot, we build two kinds of dispersal models: one based on the shortest distance to the whole afforestation (D1) and another based on the shortest distance to each individual afforestation (D2). To account for potential nonlinear trends in the inhomogeneous Poisson process, we also built dispersal models with quadratic terms of the distance to afforestations. In addition, we fitted two kind of local ground heterogeneity models: one with all uncorrelated local covariates, *H*_full_ (i.e., percentage cover of *Acaena integerrima* Gillies *ex* Hook. & Arn., *Baccharis magellanica* (Lam.) Persoon, *Festuca pallescens* (St. Yves) Parodi*, Oreopulus glacialis* (Poepp.) Ricardi and bare soil); another, *H*_reduced_ with only the covariates remaining after a stepwise selection procedure based on AIC.

Finally, in the so‐called combined models (dispersal and ground heterogeneity together), we fitted two kinds of models: the first included the covariates from the *H*_reduced_ model and the covariates of model D1. The second one included the covariates from the *H*_reduced_ model and the distance covariates of model D2. These models allowed us to estimate and simultaneously compare the effect of both the distance to afforestations and the ground plot cover on the spatial pattern of pine invaders.

### Spatial model evaluation

In addition to comparing the fitted models on the basis of their AICs, we performed a spatial model evaluation. Basically, this procedure consists in evaluating the consistency of the inhomogenous *L*‐function of each pattern with the fitted inhomogeneous Poisson process. This is similar to the usual test of Complete spatial randomness (CSR), that is, it implies computing simulation envelopes, but in this case simulating from the fitted inhomogeneous Poisson model. For this, an intensity function (i.e., an intensity surface) was estimated from each model. In the case of inhomogeneous Poisson processes, this step is totally analogous to predicting from a fitted GLM model (Baddeley and Turner 2000).

The simulation procedure comprises two steps. In a first step, a homogeneous Poisson process is simulated within the plot, and in a second step, each simulated point *x* is “thinned” with a probability proportional to 1/*λ*(*x*), where *λ*(*x*) is the intensity estimated at location *x* (Baddeley and Turner 2000). If the inhomogeneous *L*‐function shows significant deviations from the heterogeneous Poisson process at scales *r* > 10 m, we can conclude that the model does not account for the environmental heterogeneity. For each plot, we compared the inhomogeneous *L*‐function, computed on the pattern of pines, with the 3rd lowest and 3rd highest value of 99 simulations of the inhomogenous Poisson process. As in the previous analyses, we performed GOF tests to control inflation of type I errors, and additionally employed the GOF statistic as a measure of fitting: the smaller the values of the GOF statistic (i.e., the smaller the deviation from the fitted model), the better the model.

All the analyses (test and modeling of heterogeneity, and spatial model evaluation) were performed for each of three set of data: the whole pine population in each plot and the large and small pine population in each plot.

## Results

**Figure 3:**
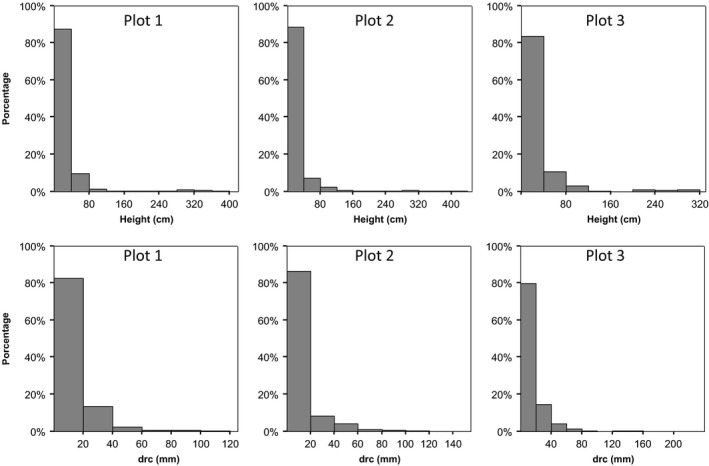
Distribution of drc (mm) and height (cm) of *Pinus contorta* individuals growing inside each of the three‐one‐hectare plots.

As expected in a Patagonian steppe, ground cover was dominated by bare soil (average values around 35%) and by the widely distributed tussock‐grass *Festuca pallescens,* with cover values ranging from 18% to 30% (Table 1, Fig. 2). The number of recruited pines was markedly different between plots, with 1006 in P1, 1142 in P2 and only 393 in P3. Concordantly, the number of small and large pines also varied between plots (168 in P1, 193 in P2 and 65 in P3 in the case of small pines and 196 in P1, 237 in P2 and 71 in P3 in large plots) (Appendix: Table A1). Independent of the density, all plots showed an inverted‐exponential recruitment curve with most pines belonging to the smallest class (Fig. 3).

**Table 1:** Ground cover for each of the three‐one‐hectare plots (100 by 100 m). Values indicate mean (% ± SD) in the 5 × 5 m subplots (*n* = 400, in each plot). Bare soil and *Festuca pallescens* dominate the steppe with a combined cover of over 50%, although there are important variations among subplots. *Baccharis magellanica*,* Oreopulus glacialis* and *Acaena integerrima* are considered cushion plants and account for ca. 5% of the ground cover

	PLOT 1	PLOT 2	PLOT 3
*Festuca*	32.06 ± 25.02	21.17 ± 17.17	18.05 ± 18.22
*Baccharis*	2.33 ± 4.04	3.00 ± 4.09	3.35 ± 4.92
*Oreopulus*	0.85 ± 1.76	0.52 ± 1.08	0.46 ± 0.76
*Acaena*	0.90 ± 1.75	0.16 ± 0.41	0.42 ± 0.74
Bare soil	39.81 ± 26.45	36.53 ± 19.23	37.68 ± 22.26
Rocks	0.29 ± 2.18	6.62 ± 14.23	7.77 ± 14.56
Others	1.30 ± 3.45	0.59 ± 1.87	0.57 ± 0.41

**Figure 4:**
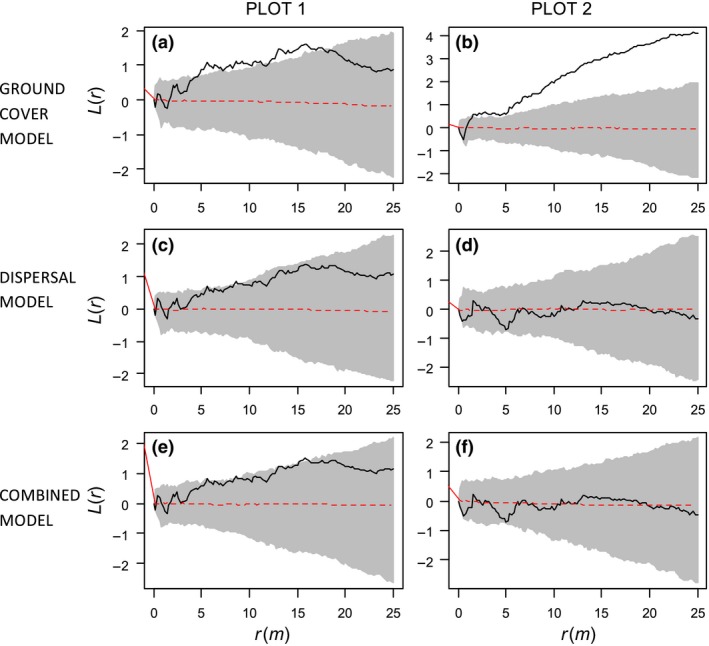
Inhomogeneous functions for large pines in plots P1 (left panels A, C, and E) and P2 (right panels B, D and F). The inhomogeneous surfaces, used to account for heterogeneity and simulate patterns for computing envelopes, were fitted by different point pattern models. Top row (panels A and B): H_full_ (i.e., models fitted to all uncorrelated ground covariates). Medium row (panels C and D): D2 (i.e., models fitted to distance to each individual afforestation). Bottom row (panels E and F): “Combined” models D2H (i.e., models including both the ground covariates from the H_reduced_ model and the distance covariates of model D2). Solid black line represents the transformed (i.e., $\sqrt{K(r)/\pi -r}$) inhomogeneous K‐function, gray bands represent 95% envelopes (computed from 199 simulations of the fitted heterogeneous model), and dashed red lines, the expected value. According to GoF tests, only the ground‐cover model for plot P2 (panel B) was unable to describe their pattern.

**Figure 5:**
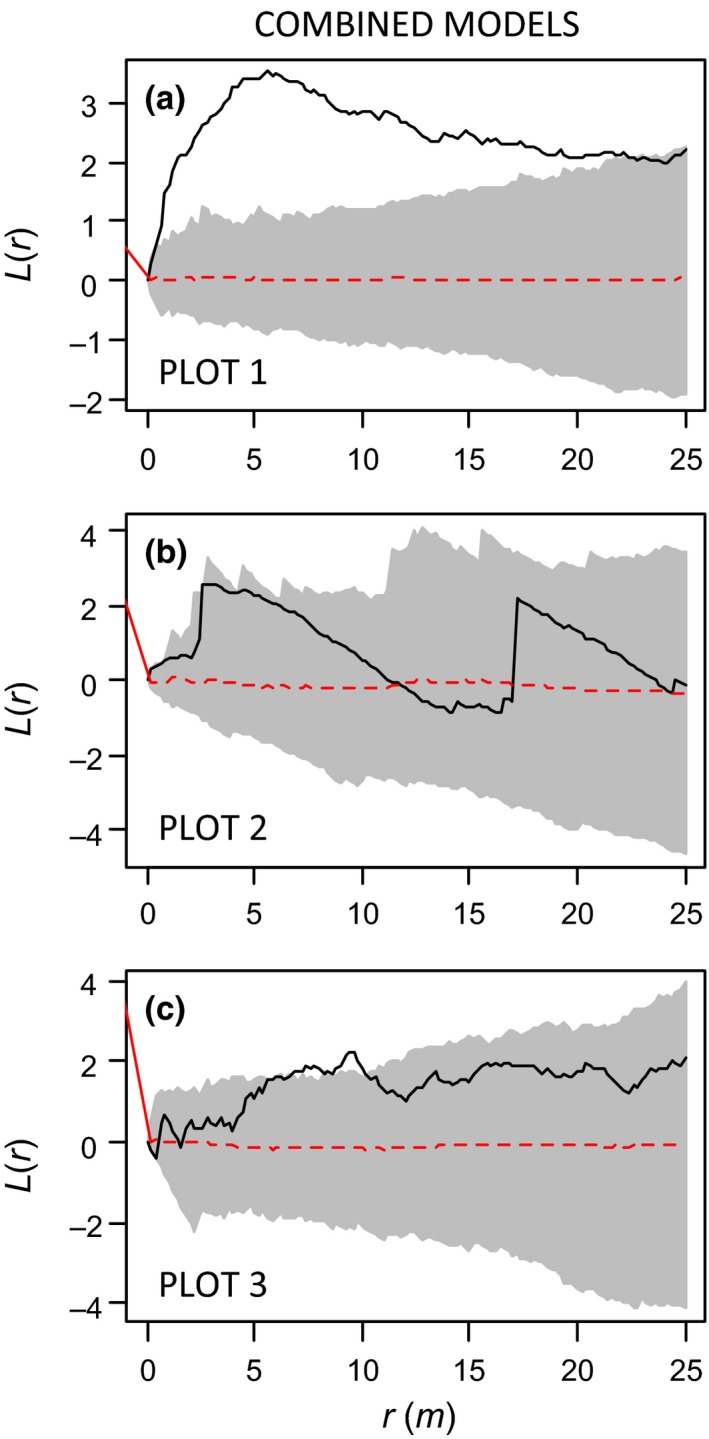
Inhomogeneous functions for small pines in plots P1 (A) and P2 (B) and P3 (C). Solid black line represents the transformed (i.e., $\sqrt{K(r)/\pi -r}$) inhomogeneous K‐function, gray bands represent 95% envelopes (computed from 199 simulations of the fitted heterogeneous model), and dashed red lines, the expected value. The inhomogeneous surfaces, used to account for heterogeneity and simulate patterns for computing envelopes, were fitted by the “combined” hmodels D2H (i.e., models including both the ground covariates from the Hreduced model and the distance covariates of model D2). According to GoF tests, this kind of model was able to describe the pattern of small pines only in plots P2 y P3 (panels B and C).

**Figure A1:**
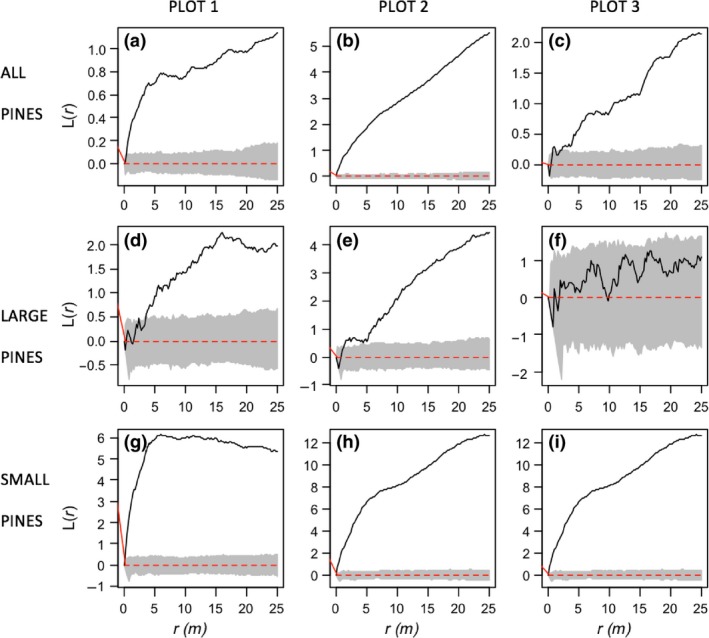
Test of heterogeneity. Solid black line represents the transformed (i.e., $\sqrt{K(r)/\pi -r}$) *K* function, grey bands represent 95% envelopes (computed from 199 simulations of CSR patterns), and dashed red lines, the expected value under CSR. From left to right, column panels correspond to plots P1, P2, and P3, respectively. From top to right, row panels correspond to total‐, large‐, and small‐pine populations. Only large pines in P3 (panel F) showed a homogeneous pattern. All the other populations were clearly heterogeneous.

As hypothesized, the spatial patterns of recruited pines showed coarse scale heterogeneity except in the case of large pines in the P3, which clearly did not depart from a homogeneous Poisson process (i.e., randomness; see Appendix: Fig. A1). We fitted inhomogeneous Poisson models to explain such large‐scale heterogeneity in the plots. In the P1, our models explained only the heterogeneity of large pines (Table 2, Figs. 4, 5). It is worth to note that nonsignificant *P* values indicate that the observed spatial patterns are not different from expectations from an inhomogeneous Poisson process. In other words, the observed departure at large spatial scales in the large tree size class can be explained by considering that the heterogeneity in dispersal and ground‐cover factors modifies the intensity of points (i.e., pine recruits). None of the fitted models correctly described the spatial structure of small pines or the whole population in P1, which means that probably other factors are controlling the realized pattern heterogeneity in these cases.

**Table 2:** Goodness‐of‐fit statistic and associated *P*‐value of models that passed the spatial model evaluation. GOF is the sum of the squared differences between the values of the observed and the mean of the simulated *K*(*r*) functions (Loosmore and Ford 2006). Smaller GOF (and larger *P*‐values) indicate smaller deviations of the observed pine pattern from the predictions of the fitted model and are indicative of better fitting. Only those models that were considered explicative in the “spatial model evaluation” step (i.e., with nonsignificant GOF) were included. Bold typed model are those with the smallest AIC for each plot and age class. R indicates those cases in which the best model is not a heterogeneous but homogeneous Poisson process (complete randomness). Italics type models indicate those “ground cover” models that arose from a stepwise incorporation of predictors

Data set	Heterogeneity models	Plot 1		Plot 2		Plot 3	
		GOF	*P*	GOF	*P*	GOF	*P*
All individuals	Dispersal 1					4.36E+05	0.32
	Dispersal 2			**2**.**54E+05**	0.23	**2**.**97E+05**	0.42
	Ground cover						
	Mixed D1					6.15E+05	0.15
	Mixed D2			3.16E+05	0.15	3.206E+05	0.33
Large individuals	Dispersal 1	3.29E+06	0.06	1.91E+06	0.13	R	R
	Dispersal 2	**1**.**60E+06**	0.15	4.10E+04	0.89	R	R
	Ground cover	8.43E+06	0.1			R	R
	Mixed D1	2.69E+06	0.07	1.65E+06	0.17	R	R
	Mixed D2	1.82E+06	0.16	**3**.**91E+04**	0.96	R	R
Small individuals	Dispersal 1						
	Dispersal 2			**9**.**20E+05**	**0**.**61**	**4**.**61E+06**	**0**.**21**
	Ground cover					*2*.*87E+07*	*0*.*07*
	Mixed D1					5.80E+06	0.06
	Mixed D2			1.04E+06	0.57	4.94E+06	0.16

For all the size classes in P2 and for the small and the whole population in P3, we found that several inhomogeneous models were able to explain the large‐scale heterogeneity of the plots (Table 2, Figs. 4, 5). As in the case of the large pines of the P1, the best models were those considering explicitly the distance to the different *P. contorta* afforestations (D2 models). This means that a Poisson process in which the dispersal trend affects the local intensity can explain the variation of density of the realized pine patterns.

Models considering only ground‐cover predictors were significant in a couple of cases (for large individuals in P1 and for small individuals in P3) (Table 2, Figs. 4, 5). Models with dispersal and ground‐cover predictors were not better than those with dispersal covariates alone for the other combinations of plots and tree sizes. Nevertheless, in those significant models where ground‐cover covariates were included, we found contrasting responses: They showed positive coefficients for bare soil cover in the case of large trees in P1, but the corresponding model presented a poor performance in the spatial model evaluation. In the case of small pines in P3, we also found significant and positive coefficients of the *Acaena* cover, whereas in P2, *Oreopolus* exerted a significant positive effect in the case of large and total individuals. On the contrary, *Festuca* cover has negative correlations in the three plots. Finally, the quadratic form of the distance to the afforestations was never included in the final models.

## Discussion

The spatial pattern of early pine invasion in our Patagonian steppe site is mainly explained by inhomogeneous Poisson processes based on a linear, negative dependence of pine recruit intensity with the distance to afforestations. In other words, early invasion pattern is compatible with the expectations of a spatial process that distributes individual pine recruits at random, with density varying as a function of a covariate (e.g., distance to afforestation in our case). This finding concurs with the idea that early invasions depend more on seed pressure than on the biotic and abiotic relationships that arriving seeds establish with the ground surface components, including plant–plant interactions.

Although colonization in most temperate tree species is clumped (i.e., positive density dependence) (Kenkel 1988), pines tend to have random recruitments in their native range (e.g., Kremer et al. 2014) where seeds may come from different sources (e.g., dead standing trees, multiple adjacent stands, isolated trees). However in our case, the process is a bit different because it appears random but heterogeneously linked to the seed rain (i.e., inhomogeneopus Poisson process). Obviously, we are aware that such clustered pattern in most temperate trees could be at least partially a consequence of the existence of clustered processes (e.g., Poisson cluster process, Seidler and Plotkin 2006) in which mother trees act as source of recruits around them. However, this mechanism is far from being operative in the earliest stages of the colonization like in our steppe as recruited trees are only beginning to produce cones (Langdon et al. 2010).

The observed pattern and its dependence on seed sources can be easily related to the fact that *Pinus contorta* is the typical example of a tree species that it is not a good competitor within its natural range but instead it is a good pioneer in treeless environments, with seed rain and canopy openness being essential for its colonization success (Ledgard 2001; Taylor et al. 2016). Thus, the treeless Patagonia steppe offers an environment with limited barriers for pine recruitment and invasion. In the native range, *Pinus contorta* recolonize areas after catastrophic disturbance especially fire, recruiting mostly in a random pattern (Kremer et al. 2014). However, seed pressure is not a limitation for colonization within its natural range because seeds are released from the pines affected by fires without appreciable variations in the high intensity of the seed rain across the burned stands (Halpern et al. 2010). Variation in recruitment densities, in these disturbed stands, is associated with biological legacies such as large woody debris, presence of light gaps, and microsite characteristics (e.g., microtopography, soil heterogeneity) (Halpern et al. 2010). In meadows and grasslands, where a process of encroachment of *Pinus contorta* is also occurring, seed pressure may be critical and pines tend to establish near the edges of the forests or as isolated satellite trees (Kremer et al. 2014). All these features suggest that colonization within its native range is based on a very efficient strategy of dispersal after disturbance, an adaptive trait that seems to be critical and extremely valuable for its successful spreading in treeless habitats worldwide.

Our results also demonstrate that this dispersal dependent pattern is found in the three invasion stages we have surveyed (Langdon et al. 2010). As we selected plots in the invasion front, pine densities were far from those that can be found in this species within its native range or in heavily invaded areas, often with values above 10,000 individuals per hectare in the native and invaded territories (Halpern et al. 2010; Langdon et al. 2010). Thus, patterns in mature stands could become different from what we found in these early invasion stages. Specifically, in the plot most recently colonized (i.e., P3, with the smallest number of recruits), the pattern of large pines does not depart from a completely random homogeneous process. We hypothesized that precisely during these early invasion stages, and due to the harsh environment, seed and seedlings would need to establish positive biotic interactions with some plant nurses. Indeed, we expected that under bare ground conditions, pine seedlings could suffer less competition but higher risk of herbivory, frost damage, summer drought‐high temperatures and desiccation. However, this was not the case as the installation of the first recruits in this plot was strictly random. Our results coincide with invasion patterns at larger spatial scales for other *Pinus* species, such as *Pinus nigra* in New Zealand, where its invasion can be modeled mostly by dispersal variables (e.g., wind velocity, seed output) and demography (Caplat et al. 2012). However, a number of factors, other than propagule pressure (i.e., biotic and abiotic), may account for *Pinus contorta* recruitment variability within and between regions (Taylor et al. 2016).

Answering how the pines will accelerate the accumulation of individuals, as the invasion progresses, is complex. However, our results would concur with the known dichotomy between the prevalent process occurring at short distances, which seems to be dominated by the seed dispersal versus distant spread where the tail of the dispersal kernel would render a more stochastic and spatially random process (Richardson and Higgins 1998; Richardson 2001; Ledgard 2003). Obviously, such patterns could be complicated as the invasion progress simply because the role of seeds dispersal from isolated fertile pines (i.e., first recruits from long distance dispersal events, Caplat et al. 2012) could be critical for the in‐filled. If true, the resulting spatial process could be described by an inhomogenous Poisson cluster process with the overlapping effect of the afforestation seed source (i.e., distance from the seed source) and the role of seeds dispersal from these older and mature recruits. As we have almost no mature pines in our stands, we have not conducted such an evaluation.

The role of background vegetation either as a facilitator or as a competitor with pines was not relevant in this study. Few of our models included some of the ground heterogeneity components as covariables for explaining realized patterns, but both AIC and GOF tests suggested that they were never better than the more parsimonious so‐called dispersal models. This highlights the fact that at the scale of the study, ground components such as *Acaena* or *Oreopolus* cover did not interfere significantly with the new recruits to affect the observed patterns. This is particularly relevant because small‐scale manipulative experiments at the plant–plant level have detected the existence of significant biotic interactions (both attractive and repulsive) between the components of the steppe community and the pine recruits (Langdon et al. 2010). Our results confirm that under a massive and continuous source of propagules, such as those produced in a commercial afforestation, facilitative and/or competitive interactions will have little effect on the outcome of the invasion. In fact, different probability of recruitment associated with contrasting microsites would be equalized and compensated throughout the almost infinite trials caused by a permanent arrival of each in microsite; in this way, even if the probability is small, some of the propagules will succeed and make different between microsites irrelevant. An additional factor that may be affecting the recruitment in our site is mycorrhizal co‐invasion, which is a requisite for successful pine establishment (Nuñez and Dickie 2014). For our Patagonia site, ectomycorrhizal fungi associated with *P. contorta* are more diverse near the plantation, but it appears that one species (i.e. *Suillus luteus*) is sufficient to ensure successful pine establishment (Hayward et al. 2015). However, how the density of ectomycorrhizal propagules (i.e. inoculum) and their species diversity affect the success of pine invasion remains unknown.

In conclusion, it appears that there is no barrier for *P. contorta* invasion in the treeless Patagonian steppe and the vegetation is showing no sign of resistance to the invasion; given enough time and a source of seeds, this pine can invade all substrates. As recruited trees start to produce seeds, new advanced foci will be created and the invasion can be catalyzed to increase density but also to reach larger distances from the original source plantation. It does not seem an easy task to control the spreading of the pine and to avoid the forest encroachment of these valuable steppe grasslands as invasions is only constraint by seed dispersal and seed source continues to increase with time as plantations age and new invasion areas start to produce seeds. Thus, pines are planted for timber production as a forest management strategy seems very difficult to avoid the spreading of the newcomer in the Patagonian steppes.

## Conflict of Interest

None declared.

## Biometric characterization of pines, Test of heterogeneity

**Table A1:** Biometric characterization of pines for the 1 ha plots. In each plot, pines with ln(drc) < mean (ln(drc)) – sd(ln(drc)) were classified as “small”, and those with ln(drc) > mean (ln(drc)) + sd(ln(drc)) were classified as “large”. All pines not classified as small or large are shown here as medium size. N = number of pines, DRC = mean diameter at root collar, Height = mean total height

	PLOT 1	PLOT 2	PLOT 3
Small pines			
N (pine ha^−1^)	196	237	71
DRC (mm)	1.5 ± 0.4	1.7 ± 0.2	1.6 ± 0.4
Height (cm)	5.4 ± 1.8	5.6 ± 1.4	5.7 ± 1.8
Medium pines			
N (pine ha^−1^)	642	712	257
DRC (mm)	9.1 ± 5.0	7.1 ± 3.9	9.3 ± 5.2
Height (cm)	15.7 ± 8.9	12.9 ± 6.2	18.2 ± 11.3
Large pines			
N (pine ha^−1^)	168	193	65
DRC (mm)	37.8 ± 19.3	80.7 ± 22.9	44.8 ± 37.4
Height (cm)	80.0 ± 87.4	81.2 ± 38.0	93.2 ± 79.2
All pines			
N (pine ha^−1^)	1006	1142	393
DRC (mm)	25.7 ± 30.2	42.7 ± 15.9	44.3 ± 21.1
Height (cm)	53.4 ± 28.0	22.9 ± 10.6	28.4 ± 13.8
